# Accuracy of lung ultrasound for the diagnosis of consolidations when compared to chest computed tomography

**DOI:** 10.1186/2036-7902-7-S1-A13

**Published:** 2015-03-09

**Authors:** P Nazerian, G Volpicelli, S Vanni, C Gigli, C Tozzetti, A Petrioli, S Grifoni

**Affiliations:** 1 Department of Emergency Medicine and 3Radiology Department, Careggi University Hospital, Firenze, Italy; 2 Department of Emergency Medicine, San Luigi Gonzaga University Hospital, Torino, Italy

## Background

Despite the emergence of evidences on the usefulness of lung ultrasound (LUS) for the diagnosis of pneumonia, to date societal guidelines still do not recommend the use of sonography to this purpose.

## Objective

Our study assesses the accuracy of LUS for the diagnosis of lung consolidations when compared to chest compute tomography (CT).

## Patients and nethods

This was a prospective study on a population complaining of respiratory symptoms of unexplained origin in the emergency department, who underwent chest CT. LUS was blindly performed to assess the diagnosis of lung consolidations, and then compared with chest CT.

## Results

We analyzed 285 consecutive patients. Chest CT was positive for at least one consolidation in 87 studies. LUS was feasible in all patients and in 81 showed at least one consolidation, with a good inter-observer agreement (k=0.83), sensitivity 82.8% (95% CI 73.2-90) and specificity 95.5% (95% CI 91.5-97.9). Sensitivity raised to 91.7% (95% CI 61.5-98.6) and specificity to 97.4% (95%CI 86.5-99.6) in patients complaining of pleuritic chest pain. In a subgroup of 190 patients who underwent also chest radiography (CXR), the sensitivity of LUS (81.4%, 95% CI 70.7-89.7) was significantly superior to CXR (64.3%, 95%CI 51.9-75.4) (p<0.05); whereas specificity remained similar (94.2%, 95% CI 88.4-97.6 vs 90%, 95% CI 83.2-94.7).

## Conclusions

LUS represents a reliable diagnostic tool alternative to CXR for the diagnosis of lung consolidations at bedside, in patients with respiratory complains.

